# Incidental detection of myeloid/lymphoid neoplasms with fibroblast growth factor receptor 1 rearrangement on fluorodeoxyglucose positron emission tomography

**DOI:** 10.1002/jha2.782

**Published:** 2023-09-07

**Authors:** Hiroki Kobayashi, Soichiro Okamoto, Mamoru Fujishima

**Affiliations:** ^1^ Department of Internal Medicine Tsuyama Chuo Hospital Okayama Japan; ^2^ Department of Haematology, Oncology and Respiratory Medicine, Okayama University Graduate School of Medicine Dentistry and Pharmaceutical Sciences Okayama Japan; ^3^ Department of Radiology Tsuyama Chuo Hospital Okayama Japan

**Keywords:** HSC transplantation, myeloproliferative disorder, PET

1

A 57‐year‐old woman with a history of endometrial carcinoma underwent a follow‐up ^18^F‐labeled fluorodeoxyglucose (FDG) positron emission tomography/computed tomography (PET/CT) scan. Although no recurrence of the endometrial carcinoma was observed, PET/CT imaging revealed diffusely increased FDG uptake in the bone marrow (Figure 1, left). Laboratory investigations revealed a white blood cell count of 46,400/mm^3^ (reference range: 3300–8600/mm^3^); a hemoglobin level of 12.3 g/dL (reference range: 11.6–14.8 g/dL); and a platelet count of 820,000/mm^3^ (reference range: 158,000–348,000/mm^3^). A bone marrow smear revealed an extremely hypercellular marrow with megakaryocyte hyperplasia, while a bone marrow biopsy found no evidence of myelofibrosis. Peripheral blood analysis did not reveal *JAK2*, *CALR*, or *MPL* mutations, and the *BCR*/*ABL* fusion gene could not be detected using molecular biology techniques. However, cytogenetic analysis revealed 46, XY, t(8;22)(p11.2;q11.2) [20/20 cells] and interphase fluorescence in situ hybridization confirmed genomic rearrangement of fibroblast growth factor receptor 1 (*FGFR1*). Based on these findings, the patient was diagnosed with myeloid/lymphoid neoplasm with FGFR1 rearrangement. Considering the poor prognosis associated with this disease [1], the patient underwent allogeneic hematopoietic stem cell transplantation from a haploidentical donor with a reduced‐intensity conditioning regimen, resulting in complete remission. Post‐transplantation PET/CT imaging confirmed the resolution of the previously observed abnormal FDG accumulation (Figure 1, right) and the patient is alive without recurrence.

**FIGURE 1:**
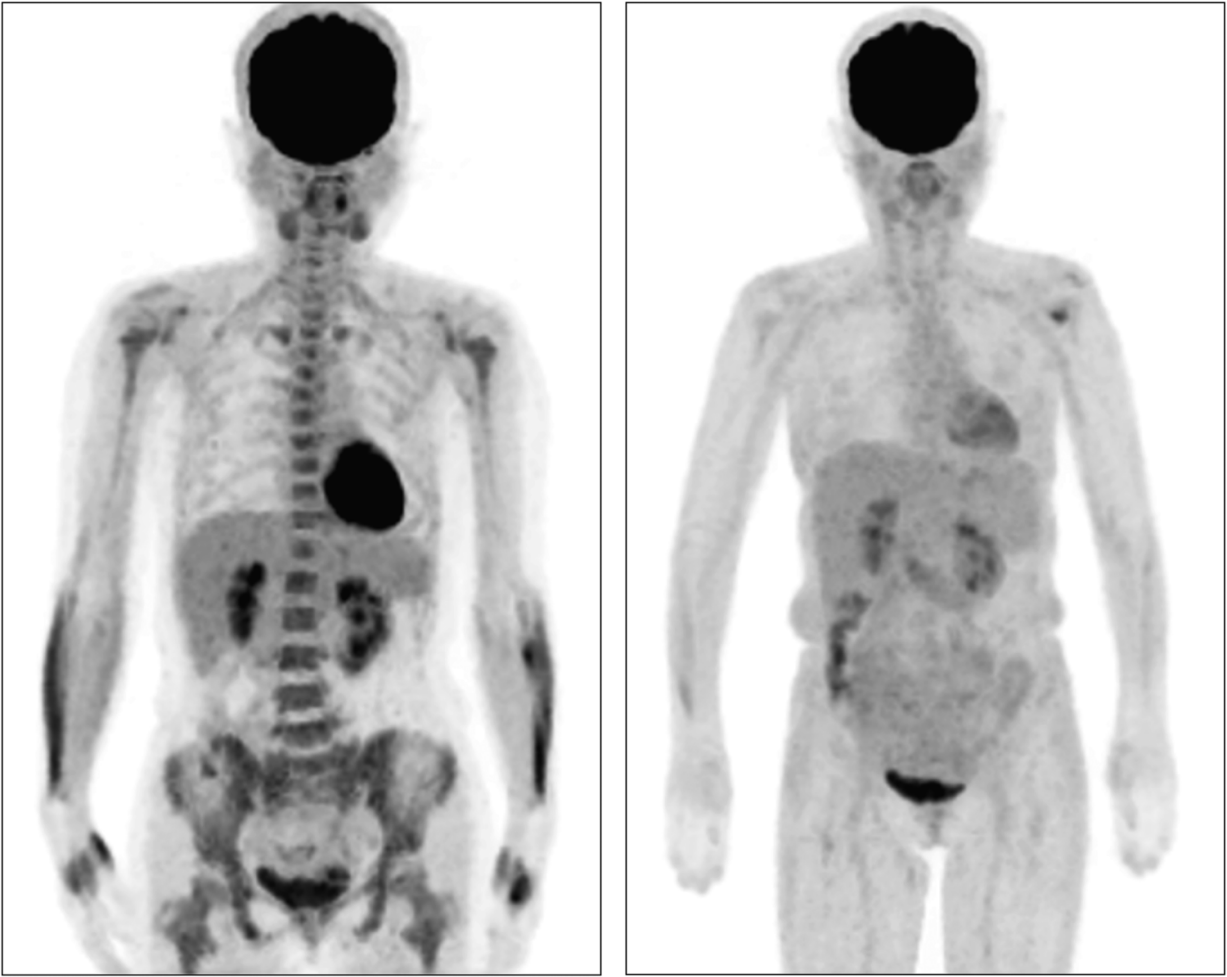
The left image shows increased FDG uptake throughout the bone marrow as demonstrated by pretreatment PET‐CT. The right image presents posttransplantation PET/CT imaging, confirming the resolution of the previously noted anomalous FDG accumulation. FDG, fluorodeoxyglucose; PET/CT, positron emission tomography/computed tomography.

PET/CT imaging is widely employed for the pretreatment staging of various tumors, including hematological malignancies. In this case, PET/CT imaging played a crucial role in accurately diagnosing a myeloid/lymphoid neoplasm with *FGFR1* rearrangement, a condition associated with a poor prognosis, enabling the patient to undergo allogeneic transplantation. Our case highlights the significance of conducting bone marrow examination and cytogenetic analysis when PET/CT imaging reveals diffuse FDG accumulation in the bone marrow.

## AUTHOR CONTRIBUTIONS

HK provided patient care and wrote the manuscript. SO and MF interpreted the PET/CT images. All authors have reviewed and approved the manuscript.

## CONFLICT OF INTEREST STATEMENT

No conflict of interest was declared.

## FUNDING INFORMATION

There were no sources of funding for this study.

## ETHICS APPROVAL STATEMENT

The authors have confirmed ethical approval statement is not needed for this submission.

## PATIENT CONSENT STATEMENT

We obtained verbal consent from the patient to publish this report.

## CLINICAL TRIAL REGISTRATION

The authors have confirmed clinical trial registration is not needed for this submission.

## Data Availability

The data supporting the findings of this study are available from the corresponding author upon reasonable request.
